# How, for Whom, and in Which Contexts or Conditions Augmented and Virtual Reality Training Works in Upskilling Health Care Workers: Realist Synthesis

**DOI:** 10.2196/31644

**Published:** 2022-02-14

**Authors:** Norina Gasteiger, Sabine N van der Veer, Paul Wilson, Dawn Dowding

**Affiliations:** 1 Division of Nursing, Midwifery and Social Work University of Manchester Manchester United Kingdom; 2 Centre for Health Informatics Division of Informatics, Imaging and Data Sciences University of Manchester Manchester United Kingdom; 3 Division of Population Health Health Services Research and Primary Care University of Manchester Manchester United Kingdom; 4 Manchester Academic Health Science Centre University of Manchester Manchester United Kingdom

**Keywords:** realist synthesis, realist review, review, virtual reality, augmented reality, simulation, training, health, health personnel, education, mobile phone

## Abstract

**Background:**

Using traditional simulators (eg, cadavers, animals, or actors) to upskill health workers is becoming less common because of ethical issues, commitment to patient safety, and cost and resource restrictions. Virtual reality (VR) and augmented reality (AR) may help to overcome these barriers. However, their effectiveness is often contested and poorly understood and warrants further investigation.

**Objective:**

The aim of this review is to develop, test, and refine an evidence-informed program theory on how, for whom, and to what extent training using AR or VR *works* for upskilling health care workers and to understand what facilitates or constrains their implementation and maintenance.

**Methods:**

We conducted a realist synthesis using the following 3-step process: theory elicitation, theory testing, and theory refinement. We first searched 7 databases and 11 practitioner journals for literature on AR or VR used to train health care staff. In total, 80 papers were identified, and information regarding context-mechanism-outcome (CMO) was extracted. We conducted a narrative synthesis to form an initial program theory comprising of CMO configurations. To refine and test this theory, we identified empirical studies through a second search of the same databases used in the first search. We used the Mixed Methods Appraisal Tool to assess the quality of the studies and to determine our confidence in each CMO configuration.

**Results:**

Of the 41 CMO configurations identified, we had moderate to high confidence in 9 (22%) based on 46 empirical studies reporting on VR, AR, or mixed simulation training programs. These stated that realistic (high-fidelity) simulations trigger perceptions of realism, easier visualization of patient anatomy, and an interactive experience, which result in increased learner satisfaction and more effective learning. Immersive VR or AR engages learners in *deep immersion* and improves learning and skill performance. When transferable skills and knowledge are taught using VR or AR, skills are enhanced and practiced in a safe environment, leading to knowledge and skill transfer to clinical practice. Finally, for novices, VR or AR enables repeated practice, resulting in technical proficiency, skill acquisition, and improved performance. The most common barriers to implementation were up-front costs, negative attitudes and experiences (ie, cybersickness), developmental and logistical considerations, and the complexity of creating a curriculum. Facilitating factors included decreasing costs through commercialization, increasing the cost-effectiveness of training, a cultural shift toward acceptance, access to training, and leadership and collaboration.

**Conclusions:**

Technical and nontechnical skills training programs using AR or VR for health care staff may trigger perceptions of realism and deep immersion and enable easier visualization, interactivity, enhanced skills, and repeated practice in a safe environment. This may improve skills and increase learning, knowledge, and learner satisfaction. The future testing of these mechanisms using hypothesis-driven approaches is required. Research is also required to explore implementation considerations.

## Introduction

### Background

As in most businesses, upskilling health care workers is vital to improving and advancing existing skills and practices and closing gaps in knowledge so that employees may continue practicing with ease [1,2]. By definition, upskilling is the process of refining existing skills or learning new skills [1]. Within the health care sector, upskilling is required to promote workforce flexibility, skill delegation, and adaptation during times of change, restructuring, or crisis [3-5]. Ultimately, this also ensures that health care delivery is safe, aligns with best practice, and is standardized across staff.

Traditional health care training consists of role modeling, shadowing, and the *see one, do one* method [6-9], along with learning through textbooks, e-learning, workshops, and seminars, as well as reading peer-reviewed journal articles. Simulation-based methods have also traditionally been used in upskilling, training, and engaging health and care providers in continued education, with the ultimate purpose of practical learning to improve patient safety [10]. These include part- or full-body manikins, synthetic latex–based simulation models, bench-top simulators, human actors, and live animal and cadaveric procedures. However, a lack of time, inaccessible resources, and a tendency to rely on experiential knowledge limit the ability to upskill [11,12]. Furthermore, training with traditional simulators is becoming difficult because limits are placed on work hours [13], and opportunities for learners to practice technical procedures on live animals and humans or cadavers are reduced because of ethical issues, commitment to patient safety, cost, and limited availability of resources [13-15].

Virtual reality (VR) and augmented reality (AR) training programs may help to overcome these barriers because they can be continuously available and used independently by learners, and they do not increase costs with use [16,17]. Akin to traditional simulation methods, VR and AR training programs enable repeated practice within safe environments away from patients and stress or time pressure [13,18,19]. VR and AR have already helped to upskill registered health care professionals on disaster response [20,21], technical and behavioral skills [7,17,22-24], and nontechnical cognitive skills [25-27].

VR is a computer-generated simulated environment in which users are immersed [28,29]. However, immersion levels can vary greatly. For example, in nonimmersive VR, environments can simply be projected onto computer screens, whereas in fully immersive VR, users wear a headset to feel as though they have been transported into a digital environment. In contrast, AR is the projection of computer-generated imagery (eg, objects) onto real-world environments [28-30], with mixed reality enabling the objects to be responsive, interactive, and spatially aware [28,29].

The effectiveness and success of VR and AR training programs is often nonlinear and complicated. This is because fidelity and perceptions of *immersion* depend on various dimensions. Fidelity refers to the extent to which an experience is close to reality [31]. Accordingly, the five dimensions that influence fidelity include physical (ie, a simulated environment), psychological (eg, stress and emotions), social, group culture, and open-mindedness of the user [32,33]. The extent to which a simulation is perceived as *good* or realistic also depends on a user’s willingness to believe in it [34]. Ultimately, this may require detail such as object collision detection (and response) or haptic technology for physical force feedback and tactile sensation [34]. These tools can introduce an additional dimension to VR by enabling users to interact with systems or manipulate digital objects through touch.

Previous literature reviews have focused on the novelty, application, and effectiveness of VR and AR training programs for health professionals, including for surgical training [13,15,18,19,35-37], nontechnical skills training [25], urology [38], disaster training [21], and dementia care [39], as well as to assess their cost-effectiveness compared with traditional simulators [40]. The reviews suggest that VR and AR may be effective for training various health care providers in both technical and nontechnical skills. However, research has also found that VR and AR training programs do not *work* for all learners, such as those who already have experience in a skill [14,41]. VR and AR learning methods are also sometimes reported as equal to, but not better than, traditional learning methods when used by nursing students [42-44] and other tools used in phlebotomy training [45]. In addition, the literature on implementing VR and AR in training for practicing health professionals is limited.

This realist review explores why there is variation in the effectiveness of VR and AR training programs and what factors influence their implementation and maintenance. Realist reviews can help to understand how, for whom, and in which contexts and conditions interventions or programs (such as the use of AR or VR for training) work. They offer a theory-driven approach to producing causal explanations of how different mechanisms of action may be triggered, which then lead to intended and unintended outcomes [46,47]. Mechanisms are changes in reasoning or individual or collective reactions (eg, behaviors, perceptions of fidelity, or cybersickness) to an intervention’s resources [46]. These mechanisms are triggered under certain circumstances, contexts, or conditions, which may relate to training scenarios, populations, or diverse AR and VR technologies.

Ultimately, a program theory developed in alignment with realist methods will result in a collection of context-mechanism-outcome (CMO) configurations that consider context, mechanisms, and outcomes. The program theory explains how an intervention may contribute to a chain of events (ie, mechanisms) that result in expected and desired or unexpected outcomes. The realist approach also considers how interventions may work differently within different contexts or conditions. CMO configurations are presented as follows:


Context + Mechanisms = Outcomes

Underlying the realist methodology is the expectation that the VR or AR intervention does not produce outcomes by itself but is instead influenced by underlying social entities, processes, or social structures (mechanisms) [46,48]. This means that it also uncovers how an intervention works in practice and results in a transferable program theory [48] that considers demi-regularities (semipredictable outcomes), which may result in varying outcomes but consistent CMO patterns [47].

### Objectives

The aim of this realist review is to develop, test, and refine an evidence-informed program theory on how, for whom, and to what extent training using AR or VR *works* for upskilling health care workers and to understand what facilitates or constrains their implementation and maintenance.

The review addressed the following questions:

1. How, for whom, and to what extent does training using AR or VR for upskilling health care workers work?
2. What facilitates or constrains the implementation (and maintenance) of training using AR or VR in health and care settings?

## Methods

### Overview

This realist review adheres to the processes explained in the RAMESES (Realist and Meta-narrative Evidence Syntheses: Evolving Standards) training documents [48]. Our protocol describes the methods in more detail [2]. In addition, we report the review in accordance with the RAMESES publication standards for realist syntheses [49]. The review followed a 3-step process, consisting of theory elicitation, theory testing, and theory refinement.

### Step 1. Elicit Theory

#### Search and Screening

The purpose of the first step was to elicit an initial program theory from candidate theories found within existing literature, which could then be refined and tested. Academic and practitioner theories were located by searching a range of databases and practitioner journals for literature on using AR or VR to upskill health professionals. The databases, search terms, and eligibility criteria are presented in Textbox 1. No constraints were imposed on the dates of publication. Learning and technology adoption theories were identified within this literature. The search was conducted between January 18 and January 25, 2021.

**Search strategy and eligibility criteria.**

**Search locations**

- Databases
  MEDLINE
  Scopus
  CINAHL
  Embase
  Education Resource Information Centre
  PsycINFO
  Web of Science

- Journals
  Academic Medicine
  MedEdPORTAL
  Medical Teacher
  International Journal of Medical Education
  Journal of Continuing Education in the Health Professions
  GMS Journal for Medical Education
  Focus on Health Professional Education
  Medical Education
  Journal of Nursing Education and Practice
  Nurse Education Today
  International Journal of Nursing Studies

**Search strategy keywords**

- Keywords with Boolean operators *AND* and *OR* (asterisk [*] indicates other variations that are covered (eg, nurs* includes *nurses*, *nurse*, *nursing*)
  *augmented reality* OR *virtual reality* AND health* OR care* OR nurs* OR *doctor* OR *surgeon* AND *training* OR *upskilling* OR *skill* OR *education* AND *evaluation* OR *implementation* OR *feasibility* OR *effectiveness*

- Search example (Scopus)
  *TITLE-ABS-KEY* (*augmented* AND *reality* OR *virtual* AND *reality*) AND *TITLE-ABS-KEY* (health* OR care* OR nurs* OR *doctor* OR *surgeon*) AND *TITLE-ABS-KEY* (*training* OR *upskilling* OR *skill* OR *education*) AND *TITLE-ABS-KEY* (*evaluation* OR *implementation* OR *feasibility* OR *effectiveness*)

**Eligibility criteria for papers identified in databases and journals**

- Inclusion criteria
  Using simulation technologies (any type of immersion)
  Health workers, care workers, and postgraduate or registered learners
  Any health, care, or university-based setting
  Covers detail on what contexts, how, and for whom they *worked* or on implementation (or maintenance)
  Published in English

- Exclusion criteria
  Simulation technologies that do not use augmentation or virtual reality (eg, web-based e-learning interventions or manikins)
  Undergraduate students
  Published in languages other than English

- Exceptions
  Work including undergraduate learners or other simulation technologies can be included if the data for postgraduate or registered learners and augmented reality or virtual reality can be separated

In alignment with previous realist reviews (eg, the study by Wong et al [50]), we conducted a 2-stage screening process, with a second researcher independently screening a random subset of papers. First, an author (NG) screened the title and abstract of each paper against the inclusion and exclusion criteria (Textbox 1) and generated a shortlist of possibly eligible papers. The full texts of these papers were then screened in the second stage. A second author (DD) independently screened a random selection of 20.2% (39/193) of the abstracts and titles and 20% (18/90) of the full texts. The raw interrater agreement rates for the 2 screening rounds were 85% and 89%, respectively. Discussion helped to reach consensus, with a third author (SNvdV) acting as a moderator.

#### Data Extraction

Data were extracted by 2 authors (NG and DD) into a coding sheet on Excel (Microsoft Corporation). This included information on the study (eg, author, date, title, research design, and sample), the intervention, contexts, mechanisms, outcomes, learning or technology adoption theories mentioned, and barriers and facilitators to implementation (or maintenance; see Table S1 in Multimedia Appendix 1). One author extracted all the data, whereas the second author reviewed 20% (16/80) of the papers for consistency. When complete CMO configurations were not provided, fragments were recorded.

#### Analysis

A narrative synthesis was conducted to determine overlapping CMO configurations and the most common barriers and facilitators to implementation and maintenance. We aggregated authors’ hypothesized mechanisms, regardless of whether they had been tested, to identify the common ways in which VR or AR affect and lead to the outcomes. The learning and technology adoption theories were also summarized and used to discuss and make meaning of the CMO configurations (in step 2).

Finally, the research team discussed the initial program theory and selected a number of CMO configurations to test, focusing on those that were expected to be most feasible, measurable, and likely to apply or transfer to future AR and VR interventions aimed at upskilling health care workers.

### Step 2. Test Theory

#### Search and Screening

The purpose of step 2 was to test the initial program theory, using existing evidence. Empirical literature was identified in a 2-step process. First, empirical studies were identified from the first search by removing nonempirical and non–full-length papers. Second, the same search as in step 1 was repeated but with a time frame of 3-6 months to identify recently published work that may have been missed. This search was conducted on March 8, 2021. We used the same screening process as in step 1 to assess the relevance of newly identified articles. The first author (NG) screened the papers to identify a shortlist of possibly eligible papers. The second author (DD) then independently screened a random selection of these papers (abstracts and titles: 2/9, 20%; full texts: 1/2, 50%), with interrater agreement rates of 100%.

#### Data Extraction and Quality Appraisal

The same items as in step 1 were extracted, along with specific evidence for the mechanisms (where applicable) and the expected outcomes identified in the initial program theory. Studies that did not provide evidence relating to the outcomes were excluded. Studies were assessed for quality using the Mixed Methods Appraisal Tool (MMAT; version 2018) [51].

The MMAT consists of 2 screening questions and 5 study design–specific criteria that could be scored 1 (yes) or 0 (no) [51]. In keeping with the studies by Pluye et al [52], Mogharbel et al [53], and Vusio et al [54], we calculated quality scores for each article and classified them as low quality (≤40%, ie, meeting 1-2 criteria), moderate quality (60%-80%, ie, meeting 3-4 criteria), or high quality (100%, ie, meeting all 5 criteria).

The quality of all the studies was assessed by 1 author (NG), whereas a second author (DD) assessed the quality of 22% (10/46) of the studies. We calculated the Cohen κ using SPSS software (version 23; IBM Corp) to determine the interrater reliability between the 2 authors.

### Step 3. Refine Theory

To refine the theory, evidential fragments (parts of studies, rather than entire studies, that provided evidence) from the second search were compared and matched to the initial program theory. We made revisions by identifying differences and presented the final theory as a narrative and diagrammatic summary. The most commonly identified learning or technology adoption theories were used to discuss the program theory.

We then assessed our confidence in each CMO configuration as high, moderate, low, or very low according to the criteria presented in Table 1. The confidence level was determined by the criterion with the lowest level. For example, if a CMO configuration had 7 supporting studies, with 4 (57%) of them contesting, and an average MMAT score of 90%, the CMO configuration was deemed *low* confidence.

**Table 1:** Criteria used to determine confidence in each context-mechanism-outcome configuration.

Confidence	Number of supporting studies	Contesting studies (if applicable), %	MMAT^a^ average score, %
High	≥8	0-20	76-100
Moderate	5-7	21-29	51-75
Low	4	30-74	26-50
Very low	≤3	75-100	0-25

^a^MMAT: Mixed Methods Appraisal Tool.

## Results

### Search Outcome

**Figure 1:**
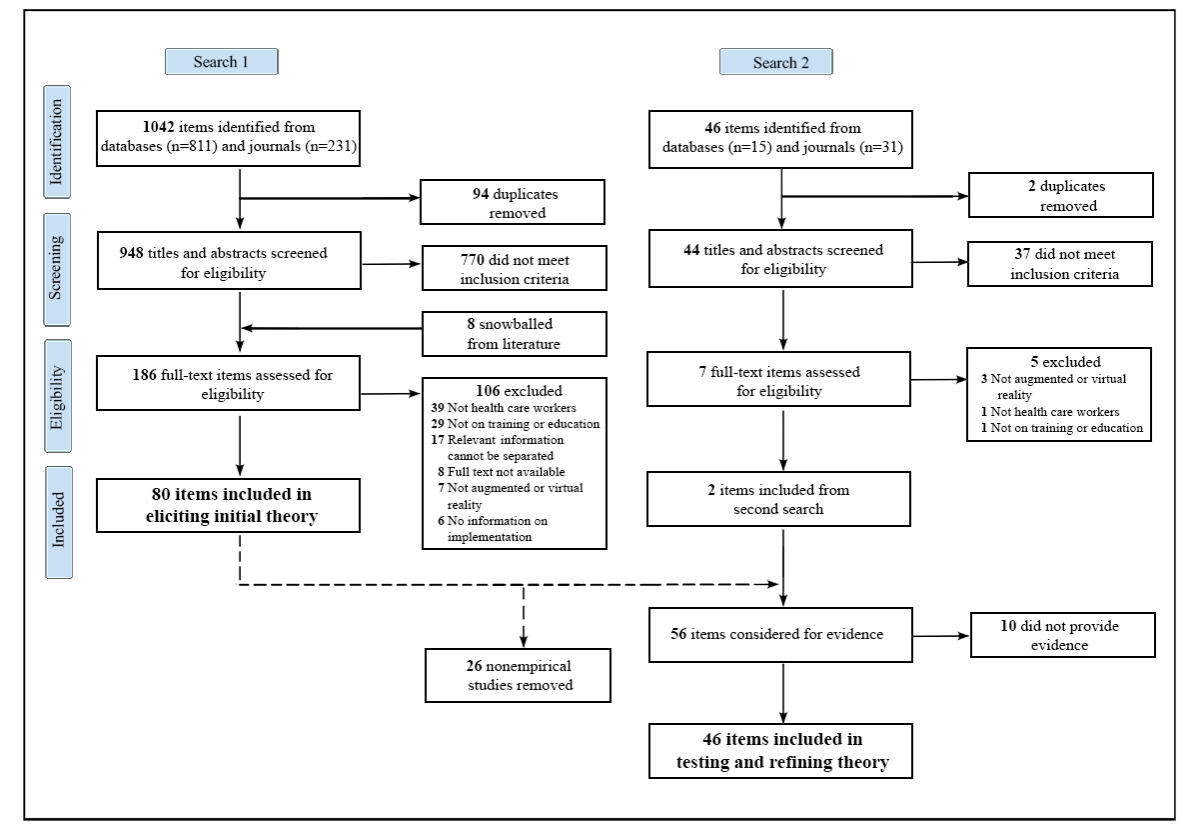
Extended PRISMA (Preferred Reporting Items for Systematic Reviews and Meta-Analyses) flowchart depicting the literature search and screening process.

The extended PRISMA (Preferred Reporting Items for Systematic Reviews and Meta-Analyses) flowchart [55] in Figure 1 shows the identification and screening process.

### Theory Elicitation

The initial search identified 1042 papers. After deduplication and abstract and title screening, 186 full texts, including 8 studies snowballed from the literature, were reviewed, of which we excluded 106 (56.9%), leaving 80 (43.01%) papers for inclusion for eliciting the initial theory. The most common reasons for exclusion were not including health care workers (39/106, 36.8%), not focusing on education and training (29/106, 27.4%), or relevant information not being separable (17/106, 16%).

### Theory Testing

The second search identified 46 recently published empirical studies. After deduplication and abstract and title screening, 7 full texts were screened, of which 5 (71%) were excluded because they did not cover AR or VR (3/5, 60%), did not include health care workers (1/5, 20%), or did not focus on education or training (1/5, 20%). Of the 7 studies, the 2 (29%) that remained were combined with the empirical literature from the first search (n=54). Of these 56 studies, 46 (82%) were included in testing and refining the theory, after 10 (18%) were excluded for not providing evidence on the CMO configurations.

### Characteristics of the Included Articles

#### Theory Elicitation

The 80 papers identified in the first search consisted of empirical research (55/80, 69%), literature reviews (22/80, 28%), case reports (2/80, 3%), and cost-benefit analyses (1/80, 1%). Of these, 83% (66/80) focused on VR, 11% (9/80) on AR, and 6% (5/80) focused on both.

#### Theory Testing

Of the 46 empirical studies included in the second stage of the review, almost half (22/46, 48%) were quantitative descriptive studies [8,9,14,22,41,56-72], 11 (24%) were randomized controlled trials [6,7,17,20,23,73-78], 7 (15%) were quantitative nonrandomized studies [16,24,79-83], 5 (11%) were mixed methods studies [84-88], and 1 (2%) was a qualitative study [89]. They were published between 1999 [80] and 2021 [59,60,83]. Of the 46 studies, 21 (46%) were conducted in the United States [7,14,17,20,22,56,58,61,63-66,72,73,75,77,78, 80-82,88], 4 (9%) in the United Kingdom [68,84-86], 3 (7%) each in China [24,67,70] and India [23,76,89], and 2 (4%) each in Germany [59,87], Taiwan [6,71], Italy [69,83], and France [57,79]. Of the 46 studies, 2 (4%) did not provide a location [8,62] and 1 (2%) study was conducted in each of the following countries: Spain [16], Canada [74], Malaysia [60], the Netherlands [9], and Australia [41].

A range of health care professionals participated, including surgeons, nurses, physicians, pharmacists, technicians, social workers, radiologists, community health workers, ophthalmologists, dentists, and respiratory therapists. Clinical experience ranged from <2 months [17] to 30 years [67]. Sample sizes ranged from 6 [24] to 109 [71] health care professionals and trainees, with an overall mean of 34.3 (SD 25.8) participants and a total of 1543 participants (of the 46 studies, 1, 2%, did not report a sample size). For those that provided a mean age, participants ranged in age from 19 years [71] to 43.7 years [87]. The characteristics of the included studies are presented in Table S2 in Multimedia Appendix 1.

### The Initial Theory

In the initial program theory, a total of 12 contexts were identified. Table 2 presents all potential CMO configurations. Informed by the initial literature screening and discussion within the research team, two contexts (1 and 6) were combined because of considerable overlap in the mechanisms and outcomes. In all, 6 contexts were chosen to be tested with empirical evidence in the next step. We had low confidence that there would be evidence available to test the remaining CMO configurations.

**Table 2:** The context-mechanism-outcome configurations identified in our initial program theory.

Context	Mechanisms	Outcomes^a^
1. Realistic (high-fidelity) simulations	Perceptions of realistic haptics and imagery Triggers interactive learning Lack of perceived realism in haptics or tactile sensation	Enhanced skills and proficiency Learner satisfaction with realism More effective learning Preference for non-VR^b^ learning, for example, laboratory dissection or physical reality
2. Artificial intelligence–enabled VR^c^	Provides feedback and highlights deficiencies	—^d^
3. VR or AR^e^ that immerses learners	Engages or exposes learners in deep immersion Provides a safe environment free from patient harm Cybersickness	Higher engagement and participation in training Improved learning, knowledge, and comfort with knowledge Improved skill performance
4. Comfortable devices^c^	Cybersickness	Poor learning experience
5. VR or AR that delivers standardized teaching	Provides feedback to leaners Enables repeated practice	Improves skill or performance Leads to better patient outcomes in the future
6. Visualization through VR or AR	Interactive experience Easier and more detailed visualization of patient anatomy Perceived realism of the imagery	Learner satisfaction with tool and realism Increased understanding or learning of content Improved performance or skill
7. Accounts for physical and mental workload^c^	Psychological improvements (reduced stress and improved self-confidence)	Decreased mental demand, effort, and physical workload scores
8. Team training delivered by AR or VR^c^	Interaction between learners and environment, as well as real-time collaboration and communication	Improves teamwork Results in learner satisfaction
9. Knowledge or skill transfer	Enhances skills Practice in safe environment (with no risk to patients) Deliberate practice	Knowledge transfer to clinical practice Skills transfer to cadaver, box trainer, and surgery and procedure Better patient care in the future
10. Used with a teacher^c^	—	Improved instruction
11. Embedded in curriculum^c^	—	—
12. Limited training opportunities	Provides feedback on performance, skill or technique Repeated practice Access to experiential learning opportunities Safe and stress-free learning environment	Skill improvement, technical proficiency, and reduced incidence of complications or errors Learner satisfaction Improvements for learners with less experience
13. Novices	Feedback and objective measurement of skills or knowledge Independent or self-directed training Safe, static, and risk-free environment without endangering patients Repeated practice Exposure to experience	Technical proficiency and skill acquisition Improved performance (including operative performance) Learner satisfaction: VR was preferred Novices (less experienced people) improved most

^a^Context + Mechanisms = Outcomes.

^b^VR: virtual reality.

^c^The context-mechanism-outcome configurations for which we had low confidence that there would be evidence available to test them.

^d^Not available.

^e^AR: augmented reality.

### Summary of the AR and VR Training Interventions

The interventions presented in the empirical literature aimed at improving technical, behavioral, or nontechnical skills. The technical skills included laparoscopic procedural skills and camera navigation [8,9,59-61,78], evacuation procedures [20], dental drilling techniques [70], and vesicourethral anastomosis during robot-assisted radical prostatectomy [24]. Nontechnical skills were less commonly focused on but included decision-making, communication, teamwork [56,85], and patient counseling and communication [89]. In keeping with the Kirkpatrick et al [90] criteria for evaluation outcomes, 78% (36/46) of the studies explored behavior or skill improvement, 67% (31/46) explored reaction to the simulators (eg, satisfaction, attitudes, opinions on user experience, or intention to use the simulator), 20% (9/46) explored knowledge or learning outcomes, and 7% (3/46) explored patient results (eg, vaccine refusal rates, patient pain, and medical errors).

Of the 46 studies, 22 (48%) used nonimmersive VR simulators, of which computer-based programs and the LapSim, AnthroSim, and MIST-VR simulators were the most commonly used [6,8,9,14,41,56,58-61,64,65,70,73,75,78,80,81,85-88]; 12 (26%) used fully immersive VR, with the most common headsets used being the Oculus Rift and HTC Vive [16,17,20,23,57,62,69,71,74,76,77,79]; and 2 (4%) used the stereographic CrystalEyes shutter glasses, which enabled 3D visualization when connected to an immersive workbench, for partially immersive VR [68,84]. Of the 46 studies, 6 (13%) used AR, with the Microsoft HoloLens glasses being the most commonly used device [7,22,66,82,83,89]. Other devices included smartphone apps, the ODG R-7 Smartglasses, and the Brother AiRScouter WD-200B headset. Of the 46 studies, 3 (7%) combined AR with VR [63,67,72]. For example, Luciano et al [63] used the ImmersiveTouch VR system in addition to high-resolution AR stereoscopic glasses. Qin et al [67] included nonimmersive VR, fully immersive VR, and AR in a comprehensive multimodal simulation training program. The study by Wang et al [24] did not clearly state the level of immersion.

Of the 46 studies, 24 (52%) used haptic technology for force feedback or tactile sensation [6,8,9,14,41,58-64,67-70, 72,73,78-80,82,84,87]. Some used other tools such as manikins [22,69,82]. For example, Semeraro et al [69] connected the commercial Laerdal HeartSim 4000 manikin to a VR headset, tracking device, and sensor gloves. Robots were also used in some studies [24,62,85] to, for example, simulate operating with the da Vinci surgical robot. Finally, a training program also used human actors who were prompted by the simulator to provide patient feedback (eg, making groaning sounds to convey pain) during endoscopy training [86].

### Quality of the Included Studies

There was substantial agreement for the MMAT appraisals between the 2 raters (NG and DD; 90%; κ=0.778, 95% CI 0.625-0.931; *P*<.001).

Overall, of the 46 studies, 13 (28%) were of high quality and 3 (7%) were of low quality, whereas the remaining 30 (65%) were of moderate quality. Of the 46 studies, 9 (20%) quantitative descriptive [8,56,58,61,63,64,66,68,70] and 4 (9%) quantitative nonrandomized studies did not include participants who were representative of the target population [24,80,82,83]; in addition, 9 (20%) quantitative descriptive studies did not clearly state their sampling strategy [41,56-58,60,62,66-68]; in 5 (11%) randomized controlled trials, randomization was not conducted properly [6,23,75-77]; in 6 (13%), blinding was either not possible or not conducted [6,7,17,20,23,76]; the qualitative approach was not reported for 2 (4%) mixed methods studies [84,85]; and the only qualitative study did not meet any of the criteria [89].

### Final CMO Configurations

**Figure 2:**
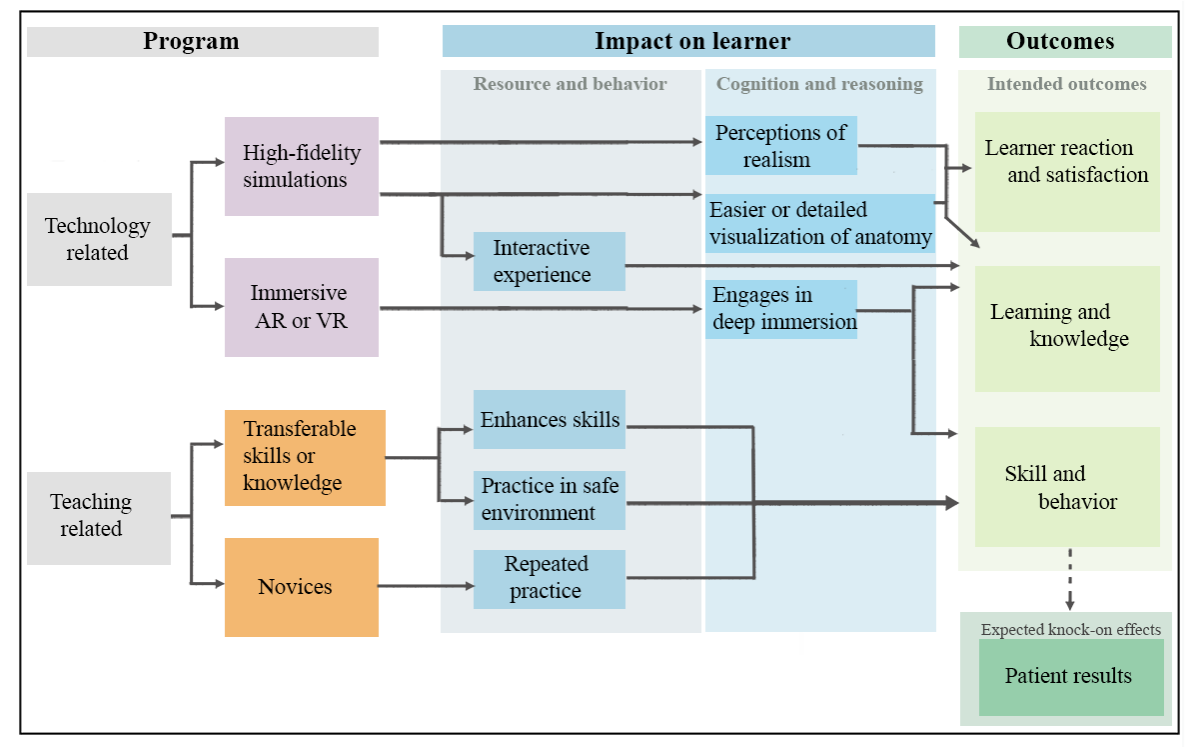
Diagram of our program theory on AR and VR training for health care workers built from the context-mechanism-outcome configurations in which we had moderate or high confidence. AR: augmented reality; VR: virtual reality.

In all, 6 contexts were identified. We distinguished technology-related conditions (Table S3 in Multimedia Appendix 1) from training-related circumstances (Table S4 in Multimedia Appendix 1). Figure 2 provides a diagrammatic summary of the CMO configurations in which we had moderate or high confidence. These are discussed in detail next. The configurations in which we had very low or low confidence are presented in Tables S3 and S4 in Multimedia Appendix 1 but without further discussion in the text.

### Realistic Simulations and Visualization (CMO 1)

The first condition relates to when VR (all levels of immersion, with and without haptics), AR, and a combination of VR and AR training programs portray realistic (high-fidelity) simulations or imagery (eg, on patient anatomy). This triggered perceptions of reality, enabled visualization of patient anatomy, and triggered an interactive experience [9,22,56,59,61,63-65,67-70, 72,79,80,82-87]. Easier visualization was explained through the use of 3D imagery, which often helped to reduce cognitive load and limit extraneous data [68,83]. The interactive experience was characterized by users interacting with the imagery in real time or when engaging in multiuser team training [56].

Across the mechanisms, 2 expected outcomes included more effective learning (increased understanding and learning of content as well as enhanced skills, proficiency, and performance) and increased learner satisfaction. There was strong supporting evidence for more effective learning when perceptions of realism and easier visualization were triggered. For example, in the study by Balian et al [22], half of the 51 participants delivered more than 80% of the cardiopulmonary resuscitation compressions with complete chest recoil and reduced leaning on the chest. This was attributed to perceptions of a realistic simulation, whereby realistic feedback included auditory (heartbeat metronome) and visual cues (increase or decrease in blood flow to vital organs). We had moderate to high confidence that easier visualization, interaction, and perceptions of realism lead to more effective learning.

Increased learner satisfaction was contested within the evidence. Some studies identified that their haptic tools hindered perceptions of realism [59,80,87]. Burdea et al [80] stated that the learners in their study were not satisfied with the VR simulator because it was not perceived as realistic. In addition, the lack of perceived realism might be why their VR group performed worse than the control group (using a rubber simulator) in diagnosing prostate cancer (33% vs 92%, respectively). It was expected that a more realistic VR simulator would have improved performance and learner satisfaction. However, most of the studies provided evidence that learners were satisfied with the tools in general [9,22,56,59,61,63,67-70,72,79,82,84-87]. We had the lowest confidence that an interactive experience resulted in learner satisfaction but moderate to high confidence that easier visualization and perceptions of realism result in satisfaction with the realism and tools, respectively.

### Immersion (CMO 2)

The second condition relates to when fully immersive VR (with and without haptics) or AR with a manikin immersed learners in the training environment [16,20,22,23,57,62,71,74,76,77,79]. This triggered perceptions of *deep immersion*, whereby learners were transported into their training environments and a safe learning environment, free from patient harm. Bhowmick et al [23] explained that isolation from the outside world and use of realistic scenarios (eg, environments, characters, and tasks) promoted feelings of deep immersion. This resulted in improved learning, knowledge, and comfort with knowledge and skill performance.

Improved learning, knowledge, and comfort with knowledge and skill performance were observed by 22% (10/46) of the studies [16,22,23,57,62,71,74,76,77,79]. For example, residents in the study by Luca et al [62] made significantly fewer major errors after the training on average (1.8 compared with 5.2). Barré et al [79] also reported decreased mental demand (thinking, deciding, and calculating) for those in the VR intervention group compared with increases in the control group. In the study by Bracq et al [57], the more users felt immersed in the environment, the more they perceived it to be useful for their learning. Increases in confidence were also observed over the training periods [23,74,76]. Given the strong evidence, lack of contrasting evidence, and the high MMAT score (78%), we had high confidence in this CMO configuration.

### Knowledge or Skill Transfer (CMO 4)

In the training-related context of knowledge and skill transfer, AR, combined AR and VR, and VR (all levels of immersion, with and without haptics) were used. When teaching transferable skills, three mechanisms may be triggered: enhancement of existing skills, practice in a perceived safe environment (away from patient harm, time restraints, and stress), and deliberate practice [14,17,20,24,41,59,71-74,77,78,81,88,89]. This leads to transfer of knowledge and skills to clinical practice and other simulators.

Empirical evidence was found for transferable skills, especially enhancing skills. Enhanced skills through VR or AR training helped to transfer knowledge and skills to clinical settings [71,88,89], other simulators (eg, sheep) [41], and surgical or invasive procedures [24,73,81]. For example, the percentage of medical and nurse trainees who experienced >1 occupational needlestick injury in the first 2 months of clinical internship was 31%-35% compared with the percentage of past senior trainees (80%) [71]. In addition, in a study by Wang et al [24], the average time required for real-life anastomosis procedures was shortened from 40.0 (SD 12.4) minutes to 25.1 (SD 7.1) minutes. However, the VR and AR simulators were not always superior and sometimes presented similar outcomes to traditional training [73,81]. In the live procedures, some medical errors (leakages) were still found [24], showing that despite improvements, performance was not perfect. We had moderate confidence that skills are transferable to clinical practice.

### Training Novices (CMO 6)

The last training-related context relates to when VR (nonimmersive and fully immersive, with and without haptics) or AR were used to train novices (learners with little or no experience). The programs were expected to trigger various resources and mechanisms, including feedback and objective measurement of skills or knowledge; independent and self-directed learning; a safe, static, and risk-free learning environment; repeated practice; and exposure to experience [6,8,9,14,17,41,58-62,65,70-72,76,79,81]. This may result in technical proficiency, skill acquisition and improved performance (including operative performance), learner satisfaction, and the most improvement in novices.

Evidence showed that repeated attempts and practice on VR or AR simulators significantly improved skills such as speed of decision-making [71], catheter-insertion depth [72], efficiency of endoscopies [58], 30° laparoscopic camera manipulation skills [60], and syringe aspiration time for central venous catheterization [65]. Given the strong supporting evidence, lack of contested evidence, and the high MMAT score (77.5%), we had high confidence that repeated practice results in technical proficiency, skill acquisition, and improved performance. Evidence for the remaining CMO configurations was very limited and often contested; thus, our confidence in them was very low or low (Table S4 in Multimedia Appendix 1).

### Implementation and Maintenance of VR and AR Training Programs

Information regarding barriers and facilitating factors for implementing and maintaining VR or AR training programs for health care professionals was extracted from the studies included in creating (step 1) and refining (step 2) the program theory.

#### Cost

Some argued that high up-front expenses created barriers to implementation and maintenance, including purchasing simulators and headsets as well as software licenses, technology maintenance, staff training, and programming requirements [26,76,91-95]. Integrating VR or AR with manikins was reported to significantly increase costs further [22]. Others argued that these costs were justified because VR can be used repeatedly at no additional cost per learner [16,17,34,40]. VR can provide a complete training tool (unlike box trainers) [60], does not wear out quickly (like manikins), and can represent any anatomy or body type, making it more cost-effective [65]. VR can also reduce time related to clinical teaching [6] and travel for trainees and educators [88].

The cost of VR and AR was expected to decrease with commercialization and market competition in this area [17,40,77], facilitating implementation as cheaper options become available [27]. A *number-needed-to-train* metric may also encourage hospital trusts and universities to implement VR programs [13]. This considers how many clinical costs each hour of training can reduce. Lohre et al [74] exemplified this metric, whereby 1 hour of training on their VR simulator was equivalent to 48 minutes of real-world training time. The simulator was therefore deemed at least 34.1 times more cost-effective.

#### Attitudes and Experience

A lack of acceptance (ie, negative attitudes) of VR and AR [56,91] and negative experiences may reduce uptake and behavioral intention [57,91,94,96]. Symptoms of cybersickness and perceptuomotor aftereffects when using VR included nausea, eye fatigue, dizziness, vomiting, and ataxia [57,91,94,96]. Other negative experiences could include addiction to VR gaming [91] and increased cognitive load and stress during initial use [57].

It was expected that a cultural change toward acceptance will occur when VR gains traction [56], which may help to increase VR as a standard teaching tool [97] and ultimately improve acceptance. Researchers have already observed positive attitudes toward these novel training tools [9,77,94,95]. For example, Ryu et al [95] reported that 81% of the 45 program directors and residents in their study expressed that VR would be a useful training tool.

#### Developmental and Logistical Considerations

Developmental and logistical considerations further create barriers because implementing and maintaining VR and AR programs requires imagination, resources, and planning [94]. From conception, the design and development of training resources can be a lengthy and complicated process, requiring specific programming and animation expertise [17,26,34,91]. Design needs to be multidisciplinary (to convey accurate content), attractive, and user centered [91]. Including external tools may further complicate development and implementation because haptic interaction systems and robotic arms may be cumbersome and limit use [61,84]. Logistical considerations also included storage space, maintenance, cleaning headsets between learners, and providing hazard-free and private learning spaces [27].

#### Access to Training

The studies highlighted access to training as a facilitator to uptake [18,27,34,40,58,62,77,98]. The mobility of AR and VR training can increase learning opportunities [18,34,62,77], which may fill educational gaps created by geographic or socioeconomic barriers [62]. Health professionals can also learn asynchronously, at their convenience [18,40], whereby self-guided training can be available to all shift workers [27,58]. These benefits also enable the potential scalability of VR and AR training [23,79,88].

Conversely, some studies reported that learners were not able to complete the training because of scheduling conflicts with patients and time constraints [27,60,95,99]. Stefanidis et al [100] clarified that initially, enthusiasm was high, but no one monitored training. Attendance only improved from 6% to 71% after a scheduling coordinator was hired.

#### Creating a Curriculum

The complexity involved in developing a standardized curriculum created barriers to implementation [8,34,37,57,91,93]. This required personnel to develop the program and schedule learners, validated training devices, and clearly defined objective criteria that aligned with existing curricula and could be used to evaluate learning outcomes [34,37,57,91,93]. Nationwide implementation was further challenged by locally established priorities, regional training budgets [93], and an unequal distribution of VR or AR resources between training centers and institutions [17,34,60].

According to the studies, leadership and collaboration are crucial to facilitate implementation [8,27,93,101]. At a local level, health professionals can develop credentialing committees [8], whereas at a higher level, national organizations and committees can help to ensure a standardized approach to training. With regard to localized training programs (eg, within hospitals), subspecialties could develop a shared training program [93,101]. Support from senior clinicians, boards of directors, and other organizational leaders is helpful to facilitate uptake [27,93,101].

## Discussion

### Principal Findings and Comparison With Prior Work

To our knowledge, this is the first realist review to explore AR and VR training programs for health care professionals. It contributes a transferable program theory that may be applicable to diverse health professionals and across AR and VR technologies with varying levels of fidelity and use of haptics or additional tools.

A total of 80 published papers were used to develop an initial program theory, and 46 empirical studies that reported on VR, AR, or mixed simulation training programs for health professionals then helped to refine and test the theory. A total of 41 individual CMO configurations were identified, across 6 contexts and conditions. Of the 41 CMO configurations, we had moderate to high confidence in 9 (22%) and low and very low confidence in 5 (11%) and 27 (59%), respectively. Our low confidence was often due to contesting studies as well as the outcomes (especially those on patient results) not being substantiated with sufficient empirical evidence.

We also identified barriers and facilitators to implementation and maintenance, which must be acknowledged for the CMO configurations to be operationalized. The most common barriers were up-front costs, poor acceptance, negative experiences (ie, cybersickness), logistics, and the complexity involved in developing a curriculum. Decreasing costs due to commercialization and the cost-effectiveness of training, a cultural shift toward acceptance, access to training opportunities, and leadership and collaboration facilitated implementation.

The CMO configurations can be explained by applying learning theories identified within some of the reviewed literature [57,83,84,87,96]. Constructivism assumes that learning is an active process, building on previous skills, knowledge and interaction with the physical and social environment [102]. Through active construction [103] and *learning by doing* [104], trainees interact with the environment to adapt and learn. In the same way, VR and AR can be used by health professionals who already have some previous experiences and acquired knowledge or skills in their clinical fields. VR and AR programs may enable upskilling through active learning by immersing health professionals within simulated *real-life* environments. This is reflected in the mechanism of immersing learners in deep immersion. The mechanisms of repeated practice, enhancing skills, and interactive experiences are also explained by constructivism because learners can interact with VR or AR environments to practice their skills.

Cognitive load theory (CLT) can also help to explain the mechanisms, especially in the context of realistic simulations and visualization. CLT assumes that people have a finite amount of working memory available [105,106]. However, we have an unlimited long-term memory, which holds cognitive schemas (experiential knowledge). Learning is then the process of constructing and automating these schemas so that it can be stored in long-term memory. Cognitive load is categorized into intrinsic load (task-specific cognitive effort), extraneous load (irrelevant cognitive effort), and germane load (residual working memory capacity).

Some of the CLT literature suggests that VR and AR may help to reduce extraneous load (ie, processes not related to learning) by providing cues and feedback in real time [68,83]. For example, AR glasses and 3D and realistic imagery can provide real-time visual clues to learning to reduce the cognitive effort of remembering this information. However, it is also possible that VR or AR learning tools may unintentionally increase task-specific or extraneous cognitive load because they may complicate learning processes. This is because learners may need to adapt to using VR or AR tools if they are not familiar with them. In some of the reviewed studies [23,57,79], health professionals reported discomfort with the VR headset because of either fatigue or cybersickness, which may also increase extraneous cognitive load because they focus on this discomfort and consequently impair their learning ability. Pretraining to gain familiarity is therefore crucial [57,107].

It was evident that the literature on implementation is premature, with little focus on implementation experiences [17,68,85,100,101]. Some of the considerations were context dependent, highlighting that when implementing VR and AR training programs, the contexts and conditions must be acknowledged. For example, novices (eg, residents and postgraduate medical and health students) may have already been exposed to VR or AR learning tools and may be more accepting of them as well as tolerant of cybersickness. This is because VR and AR is being implemented in new training curricula [77,100] and discomfort decreases with familiarity and use [79,108]. This consideration might be more relevant for those less familiar with the technologies.

### Future Research

There was a clear absence of AR and VR training programs for allied health staff, care workers, and within care- and community-based settings. There was also less focus on simple behavioral skills such as disposing of hazardous medical waste or practicing hand hygiene, for which AR and VR smartphone apps have already been developed [109]. In addition, many of the VR and AR devices were used along with haptics, robotic arms, actors, or manikins, which may introduce confounding factors when exploring effectiveness. As also identified by Kyaw et al [110], the applicability of VR or AR training within care and community settings and use as a stand-alone training tool warrants further investigation.

As is common in realist reviews [111] and evident in the literature, most of the mechanisms were not measured, except for repeated practice where authors accounted for repetitions. Control groups were rarely used, and qualitative data on experiences were limited. Future work should use robust and hypothesis-driven methods to objectively measure the impact of the mechanisms. For example, the 14-item Igroup Presence Questionnaire [112] can measure spatial presence (deep immersion), involvement (interaction), and experienced realism, whereas the 16-item Simulator Sickness Questionnaire [113] can measure cybersickness and discomfort. These validated questionnaires should be used in addition to a control group, whereas qualitative data (eg, through interviews) may help to further understand why and when the mechanisms are (or are not) triggered.

More work is also needed to increase the confidence in some of the CMO configurations for which we had low or very low confidence and to understand context-dependent implementation outcomes, along with updating the barriers and facilitators to implementation. Cost and acceptance, for example, may not be a barrier in the future, given that commercialization and market demand will reduce up-front costs, whereas increasing use may create a cultural change that favors acceptance.

### Strengths and Limitations

Unlike some realist reviews [111,114], we first used nonempirical literature to form our theory and then tested and refined it with empirical literature. This was crucial to helping us to refine the program theory; in addition, it helped to ensure that the program theory was evidence informed and more reliable. Unlike others [50], we also assessed the quality of the research used to test and refine the theory and ultimately determined our confidence in each CMO configuration. The criteria used to determine confidence were conservative and also considered contesting studies and quantity of evidence. This transparency is important because program theories developed through realist reviews are only as good as the quality and quantity of the evidence they include. To our knowledge, this is the first realist review to consider all these factors.

Limitations included not sense checking our CMO configurations with AR or VR training experts as well as not comprehensively searching for gray literature. This meant that some initial theories might have been missed. In addition, only 20% (9/46) of the included studies were assessed for quality by 2 researchers. As such, interpretation of our quality assessments may be subject to some caution. However, we did not exclude research because of low quality and amalgamated the quality of the studies to determine our confidence in the CMO configurations; therefore, we do not expect this to bias our results. Interrater reliability was also substantial.

### Conclusions

This review explored the complex nature of AR and VR training programs for health care staff, highlighting how they may actually work in practice, for whom they are most likely to work, and in which contexts and circumstances or under which conditions they may work. We found evidence for improved skills, learning and knowledge, and learner satisfaction, but there was little evidence on patient results. We had moderate to high confidence that VR and AR training programs trigger perceptions of realism and deep immersion as well as enable easier visualization of patient anatomy, interactivity, enhanced skills, and repeated practice in a safe environment. Future testing of these mechanisms using hypothesis-driven approaches is required. More research is also required to explore implementation and maintenance considerations. Ultimately, our evidence-informed program theory can be used to support the development and implementation of AR and VR training programs for health care providers and as a starting point for further research.

## Appendix

Multimedia Appendix 1 Data extraction items, summary of the empirical articles (n=46), context-mechanism-outcome configurations, and our confidence in the configurations.

## Abbreviations

AR: augmented reality
CLT: cognitive load theory
CMO: context-mechanism-outcome
MMAT: Mixed Methods Appraisal Tool
PRISMA: Preferred Reporting Items for Systematic Reviews and Meta-Analyses
RAMESES: Realist and Meta-narrative Evidence Syntheses: Evolving Standards
VR: virtual reality
