# Supplementary material for: How, for Whom, and in Which Contexts or Conditions Augmented and Virtual Reality Training Works in Upskilling Health Care Workers: Realist Synthesis
Source: JMIR Serious Games. 2022 Feb 14;10(1):e31644. doi: 10.2196/31644 (PMC8893595; doi:10.2196/31644)
Supplement: Multimedia Appendix 1 [file games_v10i1e31644_app1.docx]

**Supplementary file**

**Abbreviations**
AR: Augmented reality

CLT: Cognitive load theory

N/S: not stated

RCT: randomized-controlled trial

UK: United Kingdom

USA: United States of America
VR: Virtual reality

Table S1. Data extraction items.

| **Data items extracted** | **Explanation of items** |
| --- | --- |
| Author; date | First author surname; year published |
| Title | Full title of item |
| Type of publication | Conference paper, conference abstract, book chapter, journal article |
| Research design, theoretical orientation (if applicable) and methods | As stated |
| AR or VR technology description | Augmented reality; virtual reality; type of immersion (e.g., full or non-immersive); technology used (e.g., name of headset device); with/out haptics, short summary |
| Study objective (focus) | What the AR/VR is intended to be used for |
| Setting; country | Location covered; country (if applicable) |
| Sample (type, size, age, gender) | Sample characteristics, sample size, age and gender break-down (if applicable) |
| Context | Contexts, who used it, conditions, circumstances |
| Mechanism/s | Mechanisms that were triggered (as identified by authors) |
| Outcome/s | Intended, unintended and/or subjective outcomes, broadly related to Kirkpatrick (2007) components: 1. reaction, 2. learning, 3. behavior or 4. results. |
| Implementation | Content related to implementation and maintenance, such as strategy, adoption and/or uptake, challenges/barriers or facilitators |
| Learning or technology adoption theories | Name and summarize theories related to learning (e.g., adult learning) or technology adoption mentioned anywhere in the paper |

Table S2. Summary of the empirical articles (N=46) reviewed to test the initial program theory, and assessed for quality.

| **First author; date** | **Technology (AR/VR; level of immersion; device name; with/out haptics** | **Focus** | **Setting; Country** | **Sample; N; Mean age (range); gender (excluding medical students where applicable)** | **Kirkpatrick evaluation outcome focus** | **Learning or technology adoption theory named** | **MMAT Study Design** | **Quality (%)** |
| --- | --- | --- | --- | --- | --- | --- | --- | --- |
| Abelson; 2015 | VR; non-immersive; ICE STORM platform,  iPad and Gyration Air Mouse to interact with environment | Surgical team training | N/S; USA | Attending surgeons and trainees/residents; N=33 | 1. Reaction (opinion)  3. Skill | None | Quantitative descriptive | Moderate (60%) |
| Aggarwal; 2006 | VR; non-immersive. LapSim; haptics | Technical skills for laparoscopic surgery | N/S; N/S | Laparoscopic surgeons; N=40 | 3. Skill | Procedural skills training | Quantitative descriptive | Moderate (80%) |
| Balian; 2019 | AR; training manikin and Microsoft HoloLens glasses | Cardiopulmonary resuscitation (CPR) training | Training simulation center; USA | Healthcare providers (nurses, physicians, pharmacists, technicians); N=51. Median age 31 years; M: 15, F: 36 | 1. Reaction | None | Quantitative descriptive | High (100%) |
| Barré; 2019 | VR; full immersion; HTC Vive headset; haptics | Single-port sleeve gastrectomy training to decrease physical and mental workload | Institut Mutualiste  Montsouris; France | Surgery residents; N=10. 30.2 years; M: 6 men, F: 4 | 1. Reaction (experience)  3. Skill (performance) | None | Quantitative non-randomized | High (100%) |
| Bhowmick; 2018 | VR; full immersion; Google Cardboard headset and joystick controller | Maternal and child healthcare (labor, delivery, newborn care) | Rural health centers; India | Accredited Social Health Activists (Community Health workers); N=57; 38.2 years (27- 51); F: 57, M: 0 | 1. Reaction  2. Knowledge (learnability) | None | Quantitative RCT* | Moderate (60%) |
| Bracq; 2019 | VR; full immersion using HTC Vive headset | Surgical procedural skills training | University Hospital; France | Scrub nurses; N=13; Mean: 42 years; F: 13, M: 0 | 1. Reaction (intention to use) | UTAUT; CLT | Quantitative descriptive* | Moderate (80%) |
| Burdea; 1999 | VR; non-immersive computer-based training program; haptic (GHOST and PHANToM) | Diagnosis of prostate cancer | Medical school; USA | Urology residents; N=4. Other residents in control n/s | 3. Skill (diagnosis accuracy) | None | Quantitative non-randomized | Moderate (80%) |
| Cannon; 2014 | VR; non-immersive; ArthroSim; haptics | Knee diagnostic arthroscopy procedure | Academic institutions; USA | Postgraduate residents (year 3); N=48 | 3. Skill | None | Quantitative RCT | High (100%) |
| Charissis; 2008 | VR; partial immersion using CrystalEyes shutter glasses (learners can see others); Haptics (tracked  CyberTouch™ sensor glove) | Surgical anatomical knowledge, the principles of surgical management and operative treatment for breast cancer | NHS; Scotland | Postgraduate doctors (1-2 years clinical experience): N=12 | 1. Reaction (opinion/ experience)  2. Knowledge | Constructivist and situated learning | Mixed methods | Moderate (60%) |
| Clark; 2005 | VR; non-immersive Simbioinx GI Mentor I; haptics | GI endoscopy | Resident training program; USA | Postgraduates (year 1) and senior surgical residents; N=13 | 3. Skill | None | Quantitative descriptive | Moderate (60%) |
| Dixit; 2020 | AR; mobile app | Patient communication and counseling in ophthalmology | Ophthalmology healthcare institutions; India | Ophthalmologist trainees (10 years experience); N=45;  F: 28, M: 17 | 1. Reaction (satisfaction/opinion) | None | Qualitative | Low (0%) |
| Elessawy; 2021 | VR; non-immersive LapSim; Haptics | Laparoscopy surgical skills | Hospital; Germany | Residents, specialists  and consultants; N= 63; mean age 36; 87% male | 1. Reaction  3. Skill | None | Quantitative descriptive | High (100%) |
| Farra; 2019 | VR; full immersion; Oculus Rift headset | Hospital evacuation training for neonatal intensive care workers | Hospital; USA | NICU workers (nurses, monitor technicians,  respiratory therapists, physicians, and advanced practice nurses); N=93; 98% female | 1. Reaction  2. Knowledge  3. Skill | Bloom Taxonomy of Learning | Quantitative RCT | Low (20%) |
| Graafland; 2014 | VR; non-immersive; Simendo LCN module installed on computer; haptics | Laparoscopic camera navigation | University hospitals; Netherlands | Surgical specialists, residents, OR nurses; N=45; age means ranged from 25-42 years; gender means for the groups ranged 6-80% male | 1. Reaction (opinion)  3. Skill | None | Quantitative descriptive | High (100%) |
| Hart; 2006 | VR; non-immersive. Mentice MIST-VR; haptics | Skills when performing standard gynecologic procedures  on an ovine model | University teaching hospital and laboratories; Australia | Junior doctor trainees (years 1-3) and senior trainees (years 4-6); N=14 | 3. Skill (performance) | None | Quantitative descriptive | Moderate (60%) |
| Huang; 2018 | AR; Brother AiRScouter WD-200B AR glasses | Central venous catheter | Hospital; USA | Health professionals (doctors, respiratory therapists, sleep technicians); N=32; mean age 29.8; M: 27; F: 5 | 1. Reaction (opinion/ satisfaction)  3. Skill | None | Quantitative RCT | Moderate (80%) |
| Khan; 2012 | VR; non-immersive; Uro-mentor, Perc-mentor, Procedicus MIST-Nephrectomy and SEP Robotic simulator | Technical skills and non-technical skills  for urologists | Hospital; UK | Specialist registrars  and urological nurses; N=38 | 1. Reaction (opinion)  3. Skill (performance) | None | Mixed methods | Moderate (60%) |
| Khoo; 2021 | VR; non- immersive; LAP Mentor VRLS; haptics | Laparoscopic skills and cholecystectomy procedure | University; Malaysia | Postgraduate surgical trainees (years 1-2); N=9; Median age: 33; M: 8, F: 1 | 3. Skill (performance) | None | Quantitative descriptive | Moderate (60%) |
| Kneebone; 2003 | VR; non-immersive; VR N/S (combined with simulated patients) | Flexible sigmoidoscopy skills training | University and Hospital; UK | Nurses; N=7 | 1. Reaction (opinion)  3. Skill (performance) | Learning through community of practice | Mixed methods | Moderate (80%) |
| Koch; 2019 | VR; non-immersive; VR N/S: haptics | Trocar placement for percutaneous vertebroplasty (surgery) | University; Germany | Orthopedic, trauma surgeons and neurosurgeons; N=13; Mean age: 43.7 years; M:13, F:0 | 1. Reaction  3. Skill | CLT | Mixed methods | High (100%) |
| Kurenov; 2017 | VR; non-immersive; computer-based training program; haptics | Laparoscopic  adrenalectomy | University and Cancer Institute; USA | Residents, fellows and practicing expert surgeons; N= 28 | 1. Reaction  2. Knowledge  3. Skill | Kirkpatrick | Quantitative descriptive | Moderate (80%) |
| Lohre; 2020 | VR; full immersion; PrecisionOS platform and headset | Orthopedic surgery (reverse shoulder  arthroplasty) | Annual training course; Canada | Orthopedic surgery residents (years 4-5); N=18; mean age 31 years; 78% male | 1. Reaction (opinion)  3. Skill | None | Quantitative RCT | High (100%) |
| Luca; 2020 | VR; full immersion; Oculus Rift S headset; haptic (robotic arm) | Spine surgery | N/S; N/S | Senior surgeons and orthopedic residents/junior surgeons; N=10 | 3. Skill | None | Quantitative descriptive | Moderate (60%) |
| Luciano; 2013 | VR; non- immersive; ImmersiveTouch; haptics | Percutaneous spinal needle placement | Annual meeting and surgical training course; USA | Surgical fellows and residents; N=63 | 3. Skill (performance) | None | Quantitative descriptive | Moderate (60%) |
| Luciano; 2011 | AR/VR; non- immersive; ImmersiveTouch system and high-resolution stereoscopic display (goggles); haptics | Common neurosurgical procedure- placement of thoracic pedicle screws | Annual meeting; USA | Surgical fellows and residents; N=51 | 2. Learning  3. Skill | None | Quantitative descriptive | Moderate (60%) |
| Maytin; 2015 | VR; non- immersive; Spectranetics | Transvenous lead extraction | Hospital; USA | Incoming electrophysiology fellows; N=8 | 1. Reaction (attitudes)  3. Skill (competency) | None | Quantitative RCT | Moderate (80%) |
| McIntosh; 2014 | VR; non-immersive; computer-based training program Simbionix GI Mentor II simulator | Colonoscopy training | N/S; USA | Residents (years 2-4); N=18; mean age 29 years; M:17, F:1; | 3. Skill  4. Results (patient pain/outcome | None | Quantitative non-randomized | High (100%) |
| Ode; 2018 | VR; non-immersive; ArthroSim; haptics | Gaining  proficiency in wrist arthroscopy | Residency program in a nonprofit hospital; USA | Orthopedic residents (years 0-5); N=27.  Mean age: 29; M:24, F:4 | 1. Reaction  3. Skill | None | Quantitative descriptive | High (100%) |
| Pepley; 2017 | VR; non-immersive; custom for study | Ultrasound guided centralvenous catheteriza-  tion | Resident training program; USA | Residents and an expert vascular surgeon; N=15 | 3. Skill | None | Quantitative descriptive | Moderate (80%) |
| Pickering; 2016 | VR; non-immersive; computer program | Elder abuse and neglect recognition and reporting | Nursing agencies; USA | Nurses and social workers; N=36 | 1. Reaction (attitudes)  2. Learning (knowledge)  3. Skills | None | Mixed methods | Moderate (80%) |
| Ponce; 2014 | AR; virtual interactive presence | Orthopedic surgery | Medical center orthopedic outpatient clinic; USA | Surgical residents (years 3 and 5)  and a attending surgeon; N=7 | 1. Reaction (opinion) | Bloom Taxonomy and active participation with immediate feedback: 5- stage skill acquisition model | Quantitative descriptive | Moderate (60%) |
| Price, 2018 | VR; full immersion; Samsung Gear VR headset and smartphone | Multiple victim triage training (START model) | University; Spain | Health Professionals (enrolled in Emergency and Special Care Nursing Master's Degree); N=67; mean age 29 years | 1. Reaction (stress)  3. Skill (performance) | None | Quantitative non-randomized | High (100%) |
| Pulijala; 2018 | VR; full immersion; Oculus Rift headset and Leap Motion device | Maxillofacial  surgical technique, the Le Fort I osteotomy | Dental schools; India | Surgical residents; N=91; Mean age 27.14 years; M: 48 F: 43 | 1. Reaction (confidence)  2. Knowledge | None | Quantitative RCT | Moderate (60%) |
| Qian; 2019 | AR; ODG R-7 Smartglasses and manikin (haptic) | Neonatal intubation | NICU, hospital; USA | NICU nurses; N=45 | 1. Reaction (opinion)  3. Skill | None | Quantitative non-randomized | Low (40%) |
| Qin; 2019 | VR; non- immersive; VatsSim-XR; haptics  VR; full immersion; HTC VIVE headset  AR: partial immersion; Logitech CC2900ep HD1080p | Peg transfer training for video-assisted thoracoscopic surgery | Hospital; China | Surgical trainees (novices with 3-6 years experience and experts 11-30 years); N=32; Mean age for novices 25.5; experts 42.2 (24-56); M:28, F:4 | 1. Reaction  3. Skill | None | Quantitative descriptive | Moderate (80%) |
| Real; 2017 | VR; full immersion; Oculus Rift headset | Physician–  patient communication training to decrease vaccine refusal | Pediatric  primary care center; USA | Pediatric residents (years 2-3); N=45; M:13, F: 32 | 4. Results (vaccine refusal) | None | Quantitative RCT | Moderate (80%) |
| Sakellariou; 2009 | VR; partial-immersion; hosted on Fakespace Immersive Workbench and entails real-time visualization (CrystalEyes shutter glasses); haptics (CyberTouch™ sensor glove) | Complex anatomy training on the inguinal canal | N/S; UK | Doctor trainees and consultant level doctors | 1. Reaction (opinion of usability)  2. Knowledge  3. Skill | None | Quantitative descriptive | Moderate (60%) |
| Semeraro; 2009 | VR; full immersion; commercial Laerdal HeartSim 4000 manikin connected to VR headset and tracking device; haptics (glove) | CPR training | Congress; Italy | Medical doctors, nurses and rescuers; N=39; mean age  41.9 years; M: 27 (69.2%) F: 12 (30.8%) | 1. Reaction | None | Quantitative descriptive | High (100%) |
| Seymour; 2002 | VR; non- immersive; MIST VR system run on computer; haptics | Technical skills for laparoscopic surgery (transfer to OR) | University; USA | Surgical residents (years 1-4); N=16; M: 11, F: 5 | 3. Skill  4. Results (medical errors during operation) | None | Quant RCT | High (100%) |
| Triberti; 2021 | AR; Microsoft HoloLens | Pre-operative planning: assess patients’ anatomy (e.g., tissue to be removed) | N/S; Italy | Postgrad/Masters Medical students; N=11; mean age 24.1 (23-28 yrs); M:10, F:1 | 1. Attitude  3. Skill (performance) | CLT | Quantitative non-randomized | Moderate (80%) |
| Tsai; 2008 | VR; non-immersive; program run on desktop computer; haptics (artificial skin and Port-A-Cath) | Learning Subcutaneous infusion port injection (Port-A Cath Injection) | Hospital; Taiwan | Novice nurses; N=77 mean age 23 years; M: 0, F: 77 | 1. Reaction  2. Knowledge  3. Skill | None | Quantitative RCT | Moderate (60%) |
| Wang; 2019 | VR; immersion N/S; VR N/S and da Vinci robot | Vesicourethral anastomosis during robot-assisted radical prostatectomy | da Vinci Training Center and hospital; China | Urologists; N=6 | 3. Skill (performance) | None | Quantitative non-randomized | Moderate (60%) |
| Wang; 2015 | VR; non-immersive iDental system; haptics | Dental drilling operation | University & hospital; China | Dentists (novices, residents); N=20; M:4, F:16 | 1. Attitude  3. Skill | None | Quantitative descriptive | Moderate (80%) |
| Wu; 2020 | VR; full immersion; Headset N/S. | Needle stick (sharp injuries) training | Hospital; Taiwan | New-coming nurse and medical interns; N=109; mean age 19 and 22.76 respectively (17-29); M:33, F: 76 (70%) | 1. Attitudes (usefulness, confidence etc.)  3. Skill (performance) | Gagne flow theory | Quantitative descriptive | High (100%) |
| Yudkowsky; 2013 | AR; virtual brains;  VR; non-immersive; ImmersiveTouch; haptics | Ventriculostomy procedure training | Neurosurgery training program; USA | Neurosurgery residents (years 1-4); N=16 | 3. Skill (performance) | None | Quantitative descriptive | Moderate (80%) |
| Zackoff; 2020 | VR; full immersion; Oculus Rift headset and sensor tethered to laptop | Improving clinical reasoning & situational awareness for pediatric respiratory distress and impending respiratory failure. | Pediatric medical center; USA | Graduate nurses (most with less than 2 months clinical experience); N=48; 20-24 years; M:3, F:45 (93.8%) | 1. Attitudes (perceived effectiveness) | None | Quantitative RCT | Moderate (60%) |

*****These studies were not treated as mixed methods as they did not have sufficient information on the qualitative component.

Table S3. Technology-related conditions, mechanisms triggered, outcomes and our confidence in each CMO configuration.

| **CONTEXT** | | **MECHANISM** | | **OUTCOME** | **CONFIDENCE** |
| --- | --- | --- | --- | --- | --- |
| **Condition** | **For whom** | **Interventions** | **Response** | **Reaction, learning, skill, patient results** | **Confidence *(number of studies, contesting studies, mean MMAT)*** |
| **1. Realistic (high fidelity) simulations/ visualization** | Surgeons (residents, trainees, attendings and experts), nurses, doctors, pharmacists, technicians, postgraduate doctors, specialists, consultants, rescuers, dentists | - Combined AR and VR - AR - Fully-immersive VR with haptics - Partial immersion VR with haptics; - Non-immersive VR - Non-immersive VR with haptics | + Perceptions of realistic haptics and imagery  +lack of realism | = More effective learning (increased understanding/ learning, enhanced skills, proficiency, performance) | High  *8 studies, 0 contested, 77.5%* |
|  |  |  |  | = Learner satisfaction with tool and realism | High  *13 studies, 2 contested, 86.2%* |
|  |  |  |  | =Preference for non-VR learning e.g. lab dissection, physical reality | Very Low  *4 studies, 3 contested, 90%* |
|  |  |  | + Easier and more detailed visualization of patient anatomy | = Learner satisfaction with tool and realism | Moderate  *5 studies, 0 contested, 68%* |
|  |  |  |  | = More effective learning (increased understanding/ learning, enhanced skills, proficiency, performance) | Moderate  *7 studies, 0 contested, 71.4%* |
|  |  |  | + Interactive experience | = More effective learning (increased understanding/ learning, enhanced skills, proficiency, performance) | Moderate  *5 studies, 0 contested, 76%* |
|  |  |  |  | = Learner satisfaction with tool and realism | Low  *4 studies, 1 contested, 75%* |
| **2. VR or AR that immerses learners** | Nurses, doctors, pharmacists, technicians, community health workers, respiratory therapists, surgeons (senior, residents), postgraduate health professionals, new nurses, medical interns | - Fully-immersive VR - Fully-immersive VR with haptics - AR | + Engages learners in deep immersion | = Higher engagement and participation in training | Very low  *1 study, 60%* |
|  |  |  |  | = Improved learning, knowledge, comfort with knowledge and skill performance | High  *9 studies, 0 contested, 77.8%* |
|  |  |  | + Cyber-sickness and discomfort | =Poor learning experience | Very low  *3 studies, 2 contested, 80%* |
|  |  |  | + Provides a safe environment free from patient harm. | = Higher engagement and participation in training | Very low  *1 study, 80%* |
|  |  |  |  | = Improved learning, knowledge, comfort with knowledge and skill performance | Very low  *3 studies, 0 contested, 66.7%* |
| **3. VR or AR that delivers standardized teaching content** | Surgeons (residents, specialists), nurses (operating room, novices) doctors,  respiratory therapists, sleep technicians | - Non-immersive VR with haptics - AR | + Provides feedback to learners | = Improves skill/ performance | Very low  *2 studies, 1 contested, 70%* |
|  |  |  |  | = Leads to better patient outcomes in the future | Very low  *0 studies* |
|  |  |  | + Enables repeated practice | = Improves skill/ performance | Very low  *3 studies, 0 contested, 80%* |

Notes:
Blue highlighting identifies CMO configurations in which we have moderate or high confidence.

The mechanisms were mostly untested theories hypothesized by authors.

Table S4. Training-related conditions, mechanisms triggered, outcomes and our confidence in each CMO configuration.

| **CONTEXT** | | **MECHANISM** | | **OUTCOME** | **CONFIDENCE** |
| --- | --- | --- | --- | --- | --- |
| **Circumstance** | **For whom** | **Interventions** | **Response** | **Reaction, learning, skill, patient results** | **Confidence, (*number of studies, contesting studies, mean MMAT)*** |
| **4. When teaching transferable skills or knowledge** | Surgeons (residents, specialists), ophthalmologist trainees, nurses, monitor technicians, respiratory therapists, doctors, social workers, urologists, new nurses and medical interns | - AR - Non-immersive VR with haptics - Non-immersive VR - Fully-immersive VR - AR/VR fully immersive | + Enhances skills | = Knowledge and skill transfer to clinical practice and simulators | Moderate  *7 studies, 2 contested, 71.4%* |
|  |  |  |  | = Better patient care in the future | Very low  *0 studies* |
|  |  |  | + Practice in safe environment | = Knowledge and skill transfer to clinical practice and simulators | Moderate  *6 studies, 1 contested, 76.7%* |
|  |  |  |  | = Better patient care in the future | Very low  *0 studies* |
|  |  |  | + Deliberate practice | = Skills transfer to simulators (cadaver, box trainer^)^ and surgery/ procedure performance | Very low  *3 studies, 1 contested, 93.3%* |
| **5. When training opportunities are limited** | Residents, specialists and consultants, community health workers, nurses, social workers, electrophysiology fellows and urologists | - AR - AR with haptics - Fully immersive VR - Non-immersive VR - Non-immersive VR with haptics | + Access to experiential learning opportunities | = Skill improvement, technical proficiency and reduced incidence of complications/errors | Very low  *3 studies, 0 contested, 80%* |
|  |  |  |  | = Learner satisfaction | Very low  *3 studies, 1 contested, 66.7%* |
|  |  |  |  | = Improvements for learners with less experience | Very low  *1 study, 1 contested, 100%* |
|  |  |  | + Feedback on performance/ skill/ technique | = Skill improvement, technical proficiency and reduced incidence of complications/errors | Very low  *2 studies, 0 contested, 90%* |
|  |  |  |  | = Learner satisfaction | Low  *4 studies, 1 contested, 80%* |
|  |  |  |  | = Improvements for learners with less experience | Very low  *1 study, 100%* |
|  |  |  | + Repeated practice | = Skill improvement, technical proficiency and reduced incidence of complications/errors | Vey low  *3 studies, 0 contested, 73.3%* |
|  |  |  |  | = Improvements for learners with less experience | Very low  *1 study, 1 contested, 60%* |
|  |  |  | + Safe and stress-free learning environment | = Skill improvement, technical proficiency and reduced incidence of complications/errors | Very low  *3 studies, 0 contested, 73.3%* |
| **6. Novices** | Laparoscopic surgeons, residents/trainees, surgeons (residents, specialists and consultants), nurses, medical interns, dentists (novices, experts) | - AR - Fully immersive VR - Fully-immersive VR with haptics - Non-immersive VR - Non-immersive VR with haptics | + Feedback and objective measurement of skills/knowledge | = Novices (less experienced people) improved most | Very low  *3 studies, 0 contested, 80%* |
|  |  |  | + Independent / self-directed training | = Technical proficiency, skill acquisition and improved performance (including operative performance) | Very low  *2 studies, 1 contested, 70%* |
|  |  |  |  | = Learner satisfaction: VR was preferred | Very low  *1 study, 80%* |
|  |  |  |  | = Novices (less experienced people) improved most | Very low  *1 study, 100%* |
|  |  |  | + Learn in a safe, static & risk-free environment without endangering patients | = Technical proficiency, skill acquisition and improved performance | Very low  *3 studies, 0 contested, 86.7%* |
|  |  |  |  | = Learner satisfaction: VR was preferred | Low  *4 studies, 0 contested, 80%* |
|  |  |  |  | = Novices improved most | Very low  *1 study, 1 contested, 100%* |
|  |  |  | + Repeated practice | = Technical proficiency, skill acquisition and improved performance (including operative performance) | High  *8 studies, 0 contested, 77.5%* |
|  |  |  |  | = Learner satisfaction: VR was preferred | Low  *4 studies, 1 contested, 80%* |
|  |  |  |  | = Novices improved most | Very low  *3 studies, 1 contested, 66.7%* |
|  |  |  | + Exposure to experience | = Technical proficiency, skill acquisition and improved performance | Very low  *3 studies, 0 contested, 73.3%* |
|  |  |  |  | = Novices improved most | Very low  *2 studies, 0 contested, 70%* |

Notes:

Blue highlighting identifies CMO configurations in which we have moderate or high confidence.

The mechanisms were mostly untested theories hypothesized by authors.
